# Holistic Spatio-Temporal Graph Attention for Trajectory Prediction in Vehicle–Pedestrian Interactions

**DOI:** 10.3390/s23177361

**Published:** 2023-08-23

**Authors:** Hesham Alghodhaifi, Sridhar Lakshmanan

**Affiliations:** Department of Electrical and Computer Engineering, University of Michigan-Dearborn, Dearborn, MI 48128, USA; lakshman@umich.edu

**Keywords:** trajectory prediction, pedestrian behavior prediction, vehicle–pedestrian interaction, autonomous vehicle, connected vehicle, LSTM, graph attention network

## Abstract

Ensuring that intelligent vehicles do not cause fatal collisions remains a persistent challenge due to pedestrians’ unpredictable movements and behavior. The potential for risky situations or collisions arising from even minor misunderstandings in vehicle–pedestrian interactions is a cause for great concern. Considerable research has been dedicated to the advancement of predictive models for pedestrian behavior through trajectory prediction, as well as the exploration of the intricate dynamics of vehicle–pedestrian interactions. However, it is important to note that these studies have certain limitations. In this paper, we propose a novel graph-based trajectory prediction model for vehicle–pedestrian interactions called Holistic Spatio-Temporal Graph Attention (HSTGA) to address these limitations. HSTGA first extracts vehicle–pedestrian interaction spatial features using a multi-layer perceptron (MLP) sub-network and max pooling. Then, the vehicle–pedestrian interaction features are aggregated with the spatial features of pedestrians and vehicles to be fed into the LSTM. The LSTM is modified to learn the vehicle–pedestrian interactions adaptively. Moreover, HSTGA models temporal interactions using an additional LSTM. Then, it models the spatial interactions among pedestrians and between pedestrians and vehicles using graph attention networks (GATs) to combine the hidden states of the LSTMs. We evaluate the performance of HSTGA on three different scenario datasets, including complex unsignalized roundabouts with no crosswalks and unsignalized intersections. The results show that HSTGA outperforms several state-of-the-art methods in predicting linear, curvilinear, and piece-wise linear trajectories of vehicles and pedestrians. Our approach provides a more comprehensive understanding of social interactions, enabling more accurate trajectory prediction for safe vehicle navigation.

## 1. Introduction

**Figure 1:**
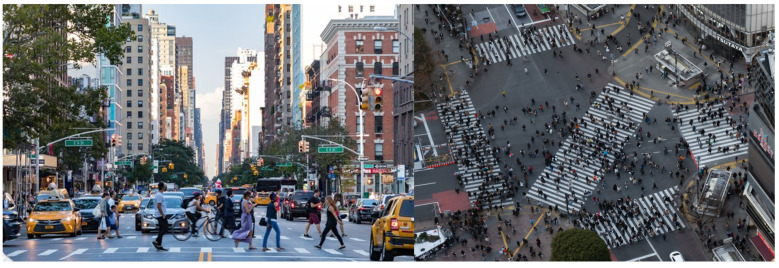
Urban environment scenarios [13,14].

Driving in an urban environment (Figure 1) is a challenging task that is associated with heavy mixed traffic flows. In a mixed traffic flow, vehicles and vulnerable road users, such as pedestrians, bicycles, and tricycles, share the same road. As a result, vehicle–pedestrian conflicts, vehicle–vehicle conflicts, and many other critical interactions regularly occur. According to U.S. National Highway Traffic Safety Administration (NHTSA) data, in 2020, 6516 pedestrians died in traffic accidents, and almost 55,000 pedestrians were injured nationwide [1].

**Figure 2:**
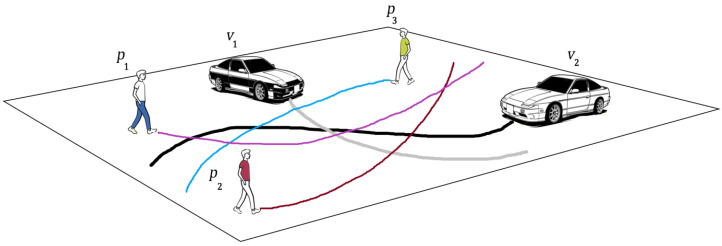
An example of vehicle–pedestrian conflicts.

The conflict between pedestrians and vehicles (Figure 2) is an important safety issue, not only in the USA but everywhere in the world. This issue is even worse in developing countries. Road accidents claim over 1.3 million lives annually, which translates to more than two lives lost every minute [2]. Shockingly, around ninety percent of these tragedies happen in countries with limited resources [2]. The sad truth is that road accidents are still the primary reason for the loss of young lives, specifically those aged 5 to 29, on a global scale [2]. For instance, in the United States, car accidents are unequivocally recognized as a principal catalyst of mortality [3]. In 2020, almost 40,000 individuals died as a direct consequence of car accidents [3]. Moreover, a considerable number, roughly 2.1 million individuals, were taken to hospital due to injuries sustained in those traffic accidents [3]. Pedestrians are among the most vulnerable road users (VRUs) because they lack the physical protection to reduce accident consequences [4]. It is not surprising that pedestrian conflicts with vehicles are most problematic in urban areas, since pedestrian activity is higher there. The problem of collisions between vehicles and pedestrians has been the subject of deep study for a long time [5,6,7,8,9,10,11,12].

The meaning of traffic conflict varies among research publications. In [15], the authors noted that operational definitions of traffic conflict could generally be categorized into two types: those based on evasive actions and those based on spatio-temporal proximity. A situation involving two or more road users, in which one user’s activity induces another user to perform an evasive move to avoid a collision, is characterized as an evasive action-based traffic conflict [16]. Pedestrian–vehicle conflicts can occur when an incoming vehicle must quickly stop or swerve to avoid a pedestrian, or when a pedestrian must take evasive action to prevent a collision. This term focuses on either the driver’s or pedestrian’s evasive actions. In contrast, proximity-based traffic conflicts are characterized as a scenario in which two or more road users are so close in space and time that there is a chance of an accident if their movements do not alter [17]. This concept suggests that the likelihood of accidents increases when road users are in close proximity to each other. Proximity can be measured using either time or space, and this conceptual definition can be put into practice by utilizing traffic detectors to measure the dimensions of time and space [18].

Numerous research studies have been conducted on conflicts between pedestrians and vehicles. However, these studies have primarily focused on examining the factors that influence such conflicts, including personal characteristics, traffic conditions, and environmental factors at crosswalks [18]. From a personal characteristics standpoint, factors such as age, gender, and disability have been investigated. For instance, the authors of [19] reported that elderly pedestrians have greater vulnerability while crossing roads as a result of a decrease in their walking capabilities. Yagil [20] identified a tendency among men to exhibit lower awareness compared to women regarding potential conflicts with vehicles when crossing roads. Tom and Granié [21] conducted an investigation focusing on gender differences in pedestrian adherence to traffic regulations, considering both signalized and unsignalized intersections. Additionally, several studies have explored factors related to traffic conditions, including variables like traffic volume and vehicle speed. Cheng [22] proposed that a high vehicle volume can lead to more severe pedestrian–vehicle conflicts because pedestrians’ protracted waiting times exceed their tolerance limits, whereas a high vehicle speed increases the chance of pedestrian–vehicle crashes. Cheng developed comprehensive models aimed at exploring the intricate associations among various variables, including pedestrian waiting time, vehicle volume, and so on. In a related study, Himanen and Kulmala [23] meticulously examined a substantial dataset consisting of 799 pedestrian–vehicle conflict incidents, ultimately identifying the most pertinent explanatory factors. These factors encompassed the distance of pedestrians from the curb, the scale of the urban environment, the number of individuals crossing simultaneously, vehicle speed, and vehicle platoon size. Additionally, researchers have extensively investigated environmental factors that contribute to pedestrian–vehicle conflicts, including city size, signal settings, road width, and lane delineation.

In the realm of autonomous vehicles (AVs), the ability to anticipate the movement of pedestrians is of paramount significance, and the consequences of neglecting it could be catastrophic. This prediction enables AVs to chart safe routes while confidently engaging in related driving tasks. Unfortunately, the intricate nature of pedestrian motion creates significant challenges for long-term trajectory prediction. It is worth noting that pedestrians’ movements are slower than those of vehicles but can change rapidly due to the complexities of human behavior. Furthermore, a pedestrian’s gait can be subjective, depending on various factors such as personal characteristics, walking objectives, and the ever-changing environment. In this dissertation, we focus on predicting the trajectory of pedestrians when interacting with other pedestrians and vehicles. Trajectory prediction is crucial for autonomous vehicles because it allows them to predict the movements of the surrounding road users several seconds into the future and make the right decision to avoid any critical conflicts. Achieving precise trajectory predictions requires the development of efficient algorithms that can accurately model and replicate real-world scenarios. Consequently, the design of such algorithms represents the most critical aspect of the task of accurate trajectory prediction.

To achieve precise pedestrian trajectory prediction, it is imperative to obtain accurate measurements. This task, however, is quite difficult due to a number of factors that can introduce inaccuracies in the collected data. These factors include occlusions caused by large vehicles and illumination issues like shadows and glare [24,25]. Additionally, pedestrians are physically smaller and lighter than most objects in their surroundings, and they can suddenly change their speed and direction, which further complicates trajectory prediction. This dissertation focuses on this challenging problem and aims to develop an efficient method for predicting pedestrian behavior via trajectory prediction. Accurate trajectory prediction assists autonomous vehicles in collision avoidance and can also be employed in smart intersections. The proposed method can also be extended to encompass the trajectory prediction of other vulnerable road users, such as bicycles, scooters, and others.

In recent years, there has been an increasing interest in developing LSTM-based methods for capturing the dynamic interactions of pedestrians. These methods utilize pooling and attention mechanisms to represent the latent motion dynamics of pedestrians in local neighborhoods or the whole scene. While pooling collects the motion dynamics of nearby pedestrians, attention assigns different importance to each pedestrian to better understand crowd behaviors based on spatial interactions. However, the temporal continuity of interactions in the crowd has been neglected in previous works. Pedestrians need to consider others’ historical movements to determine their current motion behavior and avoid potential collisions in the future, making temporal correlations of interactions important. Many other studies on predicting pedestrian trajectories have been conducted. However, most of these studies fail to take into account one of the most important factors influencing pedestrian behavior: the presence of multiple surrounding vehicles and the interaction between these vehicles and pedestrians. Although some recent studies, such as the one by Eiffert et al. [26], have attempted to incorporate such influences, they only considered a single vehicle in the presence of pedestrians. Furthermore, previous research on predicting the trajectories of heterogeneous traffic agents, such as pedestrians, has tended to focus on vehicles or motorcycles [27,28,29,30]. Additionally, it is challenging to evaluate the accuracy of pedestrian trajectory predictions due to the absence of datasets containing annotations for both pedestrian crowds and vehicles. The widely used ETH [31] and UCY [32] datasets, for example, do not include annotations for automobiles and are hence unsuitable for evaluating this task. As a result, there is a need for more research that considers the impact of various surrounding vehicles and pedestrians on pedestrian behavior, captures the spatio-temporal interactions between them, and develops more accurate algorithms for this task. Moreover, diverse datasets that contain many vehicles and pedestrians should be used to accurately investigate pedestrian trajectory prediction. To address these limitations, in this dissertation, we build a novel spatio-temporal graph attention network called Holistic Spatio-Temporal Graph Attention (HSTGA) for trajectory prediction in vehicle–pedestrian interactions, where the spatial and temporal interactions among pedestrians, as well as between pedestrians and vehicles, are encoded. Moreover, we use multiple datasets, including VCI-DUT [33], rounD [34], and uniD [35], which contain data on both pedestrians and vehicles. This enables the modeling of the influence of pedestrian–vehicle conflict on the accurate prediction of pedestrian (and vehicle) trajectories. This paper makes the following four contributions:

1. We develop a novel encoder–decoder interaction model called Holistic Spatio-Temporal Graph Attention (HSTGA) for trajectory prediction in vehicle–pedestrian interactions. HSTGA models pedestrian–vehicle interactions in non-signalized and non-crosswalk scenarios using a trajectory-based model for long-horizon pedestrian and vehicle trajectory prediction.
2. We develop a vehicle–pedestrian interaction feature extraction model using a multi-layer perceptron (MLP) sub-network and max pooling.
3. We develop an LSTM network to adaptively learn the vehicle–pedestrian spatial interaction.
4. We predict pedestrian and vehicle trajectories by modeling the spatio-temporal interactions between pedestrian–pedestrian, vehicle–vehicle, and vehicle–pedestrian using only the historical trajectories of pedestrians and vehicles. This approach reduces the information requirements compared to other learning-based methods.

## 2. Related Works

In this section, we review the existing works on the trajectory prediction of vehicle–pedestrian and vehicle–pedestrian interactions, with a special emphasis on deep learning methods.

### 2.1. Pedestrian Trajectory Prediction Methods

Over the last few years, numerous techniques and algorithms have surfaced for predicting pedestrian trajectories, owing to their importance in creating a secure environment for autonomous vehicles and other applications. The research on this topic can be broadly classified into three groups [36,37]:

1. Physics-based models.
2. Planning-based models.
3. Pattern-based models.

#### 2.1.1. Physics-Based Models

Physics-based models leverage motion properties such as speed and location to predict future movements by applying physical laws. For example, Kim et al. utilized a Kalman filter and machine learning-based approach that used velocity-space reasoning to compute the desired velocity of pedestrians, which achieved good performance [38]. Zanlungo et al. proposed a social force-based model that predicts pedestrian locations while modeling walking behaviors using the social force paradigm and physical constraints. However, the model’s performance tended to suffer when pedestrian density was low [39]. A. Martinelli et al. proposed a pedestrian dead-reckoning method that relies on step-length estimation [40]. Using classifications of walking behavior, an individual’s step length is estimated and used to infer their position. Similarly, W. Kang et al. demonstrated a smartphone-based method for pedestrian position inference that uses step-length estimation-based inference. The authors found that the method was effective in indoor environments but accrued errors over long distances [41]. Additionally, Gao et al. developed a probabilistic method for indoor position estimation that relies on Wi-Fi signal fingerprints and smartphone signals, improving accuracy and overcoming signal changes [42]. However, most physics-based models rely on manually specified parameters or rules, which limits their application to scenarios such as predicting trajectories in a closed space. In contrast, our proposed model (HSTGA) learns trajectory patterns from historical trajectory profiles without relying on manually specified parameter values.

#### 2.1.2. Planning-Based Models

In the realm of pedestrian trajectory prediction, planning-based models are typically geared toward reaching a specific destination. Ziebart et al. [43] devised a planning-based model that incorporates a distribution of destinations and utilized a Markov decision process to plan and predict trajectories. Their model outperformed a variable-length Markov model in predicting 3-second trajectories [44]. Deo and Trivedi implemented a probabilistic framework called the variational Gaussian mixture model (VGMM) [45] that utilizes trajectory clustering to predict pedestrian paths. Their model outperformed a monolithic VGMM. Rehder et al. utilized deep neural networks in their planning-based approach, inferring a mixture density function for possible destinations to conduct goal-directed planning [46]. However, this method may not perform well in long-term horizon predictions. Dendorfer et al. proposed a two-phase strategy called goal-GAN, which estimates goals and generates predicted trajectories [47]. Yao et al. improved the performance of their model by using a bidirectional multi-modal setting to condition pedestrian trajectory prediction on goal estimation [48]. Tran et al. separated their model into two sub-processes: a goal process and a movement process, enabling good performance in long-term trajectory prediction [49]. However, these models’ reliance on guessing a pedestrian’s future goals may hinder their performance in longer-horizon predictions, unlike our proposed model, which does not speculate about future goals or destinations, thus improving prediction accuracy and generalization ability.

#### 2.1.3. Pattern-Based Models

In recent years, pattern-based models have gained popularity thanks to advances in deep learning. Most studies have focused on creating modules to learn about the social features and interactions among pedestrians, which directly contribute to individuals’ movements. One notable model is the social LSTM, proposed by Alahi et al., which can predict human trajectories in crowded spaces with high accuracy [50]. It captures social interactions using a social pooling strategy to identify patterns, and it assumes that interactions among pedestrians can be captured with pooling layers in the model’s architecture. In a comparable manner, the authors of [51] implemented a distinct scaling technique to apprehend the impact of the surroundings on a particular pedestrian. Another model, social GAN, was introduced by Gupta et al., which uses generative adversarial networks (GAN) to learn about interaction patterns among pedestrians and predict their trajectories [52]. This model predicts multiple possible future trajectories and chooses the best one. Zhang et al. proposed the state refinement module, SR-LSTM, to decode implicit social behaviors among pedestrians [53], whereas Zhao et al. proposed the multi-agent tensor fusion model (MATF) to identify social and interactive relationships by aligning spatial encoding with agent encoding [54]. The multi-agent fusion model (MATF) synchronizes the spatial encoding of scenes with the encoding of each agent present within the scene and then utilizes a GAN model to acquire knowledge of patterns and make predictions. Nikhil and Morris also presented a CNN-based model that is computationally efficient and enables fast parallel processing, achieving competitive performance [55]. Huang et al. extended the temporal correlation concept to produce more socially plausible trajectories [56]. Xu et al. devised a cutting-edge methodology based on deep neural networks that harnesses the intricate nature of social behaviors to anticipate pedestrian movements [57]. The researchers deftly employ encoding schemes to distinguish the varying degrees of influence exerted by different social interactions on the trajectories of passersby. Song et al. devised a complex LSTM network that uses deep convolutional techniques [58]. The algorithm utilizes tensors to represent environmental features and incorporates a specially designed convolutional LSTM to predict sequences of trajectories. Quan et al. introduced an innovative perspective in trajectory forecasting using a model based on Long Short-Term Memory (LSTM) [59]. Their approach features a distinctive LSTM mechanism that accurately identifies pedestrians’ intentions and generates corresponding trajectory predictions. Existing models require information from all pedestrians in the scene but do not consider the impact of surrounding vehicles and the interaction between these vehicles and pedestrians on pedestrian trajectory prediction. Our approach considers these factors and uses minimal information and a decentralized method, only utilizing the pedestrian’s trajectory profile for whom the prediction is being made. The model assumes that all other factors affecting the pedestrian’s movement are unknown or uncertain, and it learns to adapt accordingly. This decentralized approach ensures that our model can provide high-quality predictions in various environments, not just crowded spaces, making it an ideal choice for practical pedestrian safety applications.

### 2.2. Vehicle–Pedestrian Interaction

Vehicle–pedestrian interactions present a critical concern in urban environments and transportation research. In the urbanization era, the safety of pedestrians has become a pressing matter. Academic studies have delved into various aspects of this complex dynamic, investigating pedestrian behavior, driver awareness, and the impact of built environments on interaction patterns. Scientists have utilized advanced approaches, such as observational investigations, simulation techniques, and data-centric analyses, to untangle the complexities of these interactions. The various findings have emphasized the significance of certain factors, such as pedestrian visibility, crossing behavior, and driver response times, in determining the safety outcomes of such encounters. Understanding these interactions is instrumental in devising effective strategies to minimize collisions and enhance pedestrian safety in our cities. As autonomous vehicles become more prevalent, ensuring seamless and safe interactions between autonomous vehicles and pedestrians assumes paramount importance. Scholars have investigated the challenges inherent in developing algorithms that can accurately predict pedestrian behavior and adapt to the dynamic nature of urban environments. The integration of cutting-edge sensor technologies, such as LiDAR and computer vision, has endowed autonomous vehicles with enhanced perception capabilities, enabling them to adeptly discern their surroundings and anticipate pedestrian actions. However, the intricacies of pedestrian behavior and the diversity of pedestrian actions continue to pose significant obstacles. Researchers have sought to address these challenges by employing machine learning techniques and reinforcement learning algorithms to enhance pedestrian detection, recognition, and trajectory prediction. The future of autonomous vehicle–pedestrian interaction rests on the successful integration of advanced AI technologies and comprehensive research insights to ensure a safer and more efficient transportation landscape. The coexistence of a dynamic vehicular entity in proximity to a pedestrian has been demonstrated to exert a substantial influence on pedestrian dynamics. Consequently, it constitutes a critical consideration in the process of pedestrian trajectory modeling and prediction [26,60]. The vehicle–pedestrian interaction has been the subject of diverse modeling approaches in the extant literature, contingent upon the employed trajectory generation model, which may encompass expert-driven or data-informed methodologies [60]. From a holistic standpoint, the interaction effects between vehicles and pedestrians can be classified into two main categories: explicit and implicit modeling.

#### 2.2.1. Explicit Interaction Modeling

In explicit interaction modeling approaches, the influence of a vehicle on a pedestrian’s dynamics is directly incorporated through explicit terms within the formulation of the pedestrian’s movement [61,62,63]. An illustration of this can be observed in the utilization of explicit forces, as presented in the social force model, where the vehicle’s effect on the pedestrian’s trajectory is explicitly represented [64,65]. The authors of [66] categorized explicit modeling approaches into four methods, namely repulsive forces, the social force model (SFM) with other collision-avoidance strategies, direct coupling of motions, and other methods.

In the repulsive forces method, the original social force model (SFM) was proposed by Helbing and Molnar [67]. The focus in the original model was on pedestrians’ social interactions. However, subsequent work has extended this model to incorporate pedestrian–vehicle interactions [68,69]. These extensions propose additional forces to account for such interactions. In these extended models, each vehicle imposes a distancing effect on pedestrians, considering their relative proximity and direction. The impact of the relative interaction distance is encompassed in what is commonly referred to as the decaying function [70]. Typically, this function is chosen as an exponential decay based on the distance [71,72]. An additional component incorporated into certain formulations of social force models (SFM) is the anisotropy function [70,73]. This function accounts for the impact of various interacting directions on the strength of the repulsive force. As an example, the model considers that a pedestrian approaching a vehicle will experience a greater impact than another pedestrian moving away from the vehicle [70,73]. Certain works have employed circular representation for vehicles, similar to the modeling of pedestrians in SFM, but with a notably increased radius [64]. Different models have been proposed to account for the danger zone around a vehicle and the interaction force experienced by pedestrians. Some models use an ellipse with one focus at the rear of the vehicle and the other extended depending on the vehicle’s speed [71]. Other models use a fixed ellipse or a rectangular shape contour to enclose the vehicle, with the magnitude of the repulsive force adjusted based on the distance and orientation of the pedestrian [70].

The second method in explicit modeling is the social force model in combination with other collision-avoidance methods [61,64,72,74]. In this approach, the SFM is combined with other collision-avoidance strategies to handle potential collisions and conflicts. In [61,72], a long-range collision-avoidance method was proposed to predict conflicts by projecting the pedestrian’s shadow and calculating the minimum speed and direction change to avoid a collision. In [74], the authors presented a force that is defined to keep the pedestrian in a safe zone by modeling their tendency to walk parallel to the vehicle. In [64], a decision model based on the time-to-collision parameter was used alongside the SFM to determine actions for different types of interactions with a vehicle. The capability of the SFM to seamlessly link perception with action was effectively applied in [62,75,76] to address straightforward reactive interactions. Nonetheless, to tackle more intricate interactions involving decision making among multiple alternative actions, an additional game-theoretic layer was introduced above the SFM.

The third method in explicit modeling is the direct coupling of motions approach. Modeling the interactions can involve coupling the motion equations of both agents, taking into direct account the impact of an agent’s actions on the motion decisions of the other. Zhang et al. utilized a constant turn rate and velocity model (CTRV) to represent the vehicle’s motion [77]. In this proposed method, a correlation between the state of the pedestrian and the coordinate system of the ego vehicle was created. Additionally, alternative approaches exist that explicitly consider the vehicle’s influence on pedestrians’ future states. In [78], the pedestrian’s speed and direction are selected at each time step to ensure a collision-free trajectory when their paths intersect with the vehicle. In [79], the impact of the vehicle on the pedestrian’s velocity is considered by incorporating an assessment of the collision risk. In [64,65], Time to Collision (TTC) was applied along with the social force model to track vehicle–pedestrian interactions. In [80], a factor of collaboration pertaining to pedestrians was introduced. This factor stands as a manifest interaction component delineating the relationship between a pedestrian and a nearby vehicle.

#### 2.2.2. Implicit Interaction Modeling

Conversely, the implicit interaction modeling approach leverages the vehicle’s trajectory as an additional input to the model along with the target pedestrian’s trajectory data [30,81]. These models are usually trained on real-world scenario datasets, which helps the models learn vehicle–pedestrian interactions from these scenarios. Various approaches have been suggested for integrating the trajectories of distinct agents within the interaction module. These approaches encompass techniques like pooling mechanisms or utilizing graph neural networks. Some papers that focus on predicting the trajectory of a single pedestrian from the egocentric view of a moving vehicle try to account for the interaction between the pedestrian and the ego vehicle, using some moving features from the vehicle in the data-driven prediction model [59,82,83,84,85]. The interaction formulation in each of these three models is discussed in the following subsections. Based on the literature [59,66,82,83,85], the implicit modeling of vehicle–pedestrian interaction can be divided into three models, namely the pooling model, graph neural network model, and ego vehicle–pedestrian interaction model.

- Pooling Models

In [81,86,87], an occupancy grid map is constructed using the target vehicle’s or pedestrian’s position as its center. This map is then employed to aggregate the hidden states of all adjacent agents. Within these occupancy maps, the concealed state of all agents situated within the same grid cell is aggregated. This process constructs a tensor that encapsulates data regarding all collaborative agents capable of influencing the forthcoming trajectory of the pedestrian under consideration. Subsequently, this tensor is employed in conjunction with the spatial latent state of the target agent as the primary input for the LSTM network utilized in the trajectory prediction process. In [86], Cheng et al. introduced a circular polarization occupancy representation. This method utilizes the orientation and distance of the agents relative to the target pedestrian to define the cells that are considered occupied. In [88], a comprehensive iteration of these spatial feature maps was proposed. This is accomplished by partitioning the bird’s-eye view of the scenario into distinct grid cells. Within this map, the feature representation of each agent is seamlessly incorporated into a tensor, which accounts for the precise agent placement. Subsequently, the two-dimensional tensor at each sequential time instant is channeled into a convolutional neural network (CNN) architecture. Concurrently, a distinct LSTM architecture is employed to examine the temporal interdependencies among these spatial maps as they evolve over time. In [27], a dual-map approach was proposed for each agent, involving horizon and neighbor maps that encompass prioritized interactions and neighboring agents’ embedding, respectively. These maps are processed using convolutional neural networks and their outputs are combined with the target agent’s embedding to predict the agent’s future trajectory [27,28].

B. Graph Neural Network Model

In graph neural networks, spatial edges model the interaction between agents and their effect on future positions, using message-passing and attention mechanisms to encode the importance of connected edges. The act of extracting information from interconnected nodes in a graph and using it to enhance the representation of the node is known as message propagation. This approach finds application in defining the influence of interacting entities on a target pedestrian’s dynamics within graph neural networks (GNNs). Usually, these frameworks employ an attention mechanism to capture the proportional importance of connected edges concerning the specific agent of interest. In [30,89,90], a widely accepted criterion was introduced centered on spatial separation. This criterion entails establishing a link between two agents in a graph, defined as a spatial edge, when their proximity reaches a specified threshold distance. Although certain articles employ a set criterion to determine connected edges, others opt to initiate with a completely connected graph [91]. In simpler terms, this entails considering all agents present within the scene. In [92], a reinforcement learning approach was used to investigate the existence of these edges between agents. Actions entail switching the state of each edge on or off, while rewards are based on the overall accuracy of trajectory predictions associated with the particular graph link. Several studies have employed directed graphs instead of undirected versions to address interaction asymmetry [89,91,93,94,95]. The authors of [89] employed encoded interactions in a graph-based context to predict the short-term intentions of agents using a probability distribution function. Then, this predicted intention, in conjunction with the inherent graph arrangement, facilitates the future trajectory for individual agents. Several scholars have employed the graph convolutional network (GCN), applying it directly to graphs. They formulate an adjacency matrix to represent connections within the graph, where the matrix’s weights reflect the reciprocals of agents’ relative speeds or distances [30,94,96,97]. Other researchers have proposed alternative GNN techniques that utilize recurrent neural networks (RNNs), such as LSTMs, to capture the time-evolving characteristics of the edges within the graph [93,98,99].

C. Ego Vehicle–Pedestrian Interaction Model

Typically, these interactions are represented by incorporating certain attributes of the ego vehicle’s movement along with the positional sequences of the pedestrian. One common attribute employed for this purpose is the speed of the ego vehicle, which can significantly influence the choices and movement of the pedestrian engaged in the interaction. In [59,82,83,84,85], the speed of the ego vehicle was employed to anticipate the subsequent actions of the pedestrian within the camera’s image. Certain proposals have arisen that advocate the utilization of a separate network to forecast the future speed of the ego vehicle. This projected speed can then be employed in predicting the trajectories of pedestrians [82,83]. Additional studies have incorporated elements such as the pedestrian’s relative distance from the ego vehicle [100] or the geographical coordinates of the host vehicle’s location [101] in combination with the motion attributes of other pedestrians. Kim et al. extended this approach by incorporating the pedestrian’s viewpoint [102]. They considered interaction aspects such as the relative positioning of the pedestrian and the vehicle, the orientation of the pedestrian’s head in relation to the vehicle, and the speed of the vehicle. Nonetheless, observing the scenario through the view of an ego vehicle entails that the motion sequences of all pedestrians discussed in the aforementioned works are in relation to the relative positions. Hence, incorporating vehicle attributes as an additional input to the model serves as a method for compensating for the influences of a moving frame, rather than exclusively a factor related to interactions within the model.

In brief, the modeling of interactions between vehicles and pedestrians is typically an intricate undertaking, and this intricacy is amplified in road settings lacking well-defined lanes, crosswalks, and strict traffic protocols [103,104]. In [105], the authors found that there are substantial differences in pedestrian movement patterns between structured and unstructured roads [105]. Limited research has been conducted on the interaction between pedestrians and vehicles in trajectory prediction on unstructured roads. Previous works have mostly focused on social interactions among pedestrians [50,52,106] and interactions with the environment [51,107,108]. However, the interaction between pedestrians and vehicles is an equally important factor that needs to be considered. Some researchers have tried to include vehicle information in pedestrian trajectory prediction, but their methods have limitations. Eiffert et al. [26] improved pedestrian trajectory prediction by encoding interactions between pedestrians and a single vehicle using a feature learning network called the “Graph pedestrian–vehicle Attention Network”. However, this method only considers a single vehicle on the road, not multiple vehicles. On the other hand, Chandra et al. [27,28,29] and Carrasco et al. [30] proposed models that can predict the trajectories of heterogeneous traffic agents, including pedestrians, but their primary focus was on vehicles and motorcycles rather than pedestrians. Therefore, there is still a need for more research on the interaction between pedestrians and vehicles in trajectory prediction.

### 2.3. Intelligent Vehicle Trajectory Prediction

In the realm of predicting vehicle movements, it has become increasingly evident that a more comprehensive approach is essential. The integration of perception systems, cameras, and intelligent vehicular systems has simplified the acquisition of data from both driving agents and the environment. Nevertheless, relying solely on a traffic agent’s trajectory history for prediction can result in errors, particularly in intricate scenarios. Real-life driving situations are inherently complex, and classical methods of predicting intelligent vehicle trajectories possess limitations. These methods struggle to encompass the multifaceted ways vehicles interact with their surroundings, especially concerning other road users like pedestrians, cyclists, and fellow drivers. Recognizing the significance of comprehending and modeling the diverse interactions on the road proves vital for accurate trajectory prediction. Approaches that are mindful of interactions, acknowledging inter-agent dynamics and behavioral dependencies, contribute to elevated prediction accuracy [109]. Such approaches facilitate the gathering of extensive data on the behaviors and intentions of various road users. Expanding upon the foundation of interaction-aware trajectory prediction, the utilization of graph-based interaction reasoning employs graphs to more effectively capture the intricate relationships and interdependencies between road users. This proves particularly valuable in scenarios where conventional prediction models fall short, such as navigating complex intersections, unstructured road environments, and bustling urban settings characterized by a mix of user behaviors. As cited in [109], intelligent vehicle trajectory prediction models can be categorized into two primary types: interaction-aware trajectory prediction and graph-based interaction reasoning. Our decision to follow this categorization stems from a resolute intention to enhance the fidelity, precision, and adaptability of these models.

#### 2.3.1. Interaction-Aware Trajectory Prediction

Numerous studies have endeavored to enhance interaction awareness for trajectory prediction approaches by modeling inter-agent correlations among all agents in a driving scene. The early literature on interaction awareness employed traditional approaches, such as classical machine learning models, for example, Hidden Markov Models (HMM), Support Vector Machines (SVM), and Bayesian networks [110,111,112,113]. Nevertheless, these conventional methodologies exhibit suboptimal performance in long-term predictions, particularly for intricate scenarios, and are ill-suited for real-time analysis [114].

The employment of deep learning models, specifically recurrent neural networks (RNNs), temporal convolutional neural networks (CNNs), and graph neural networks (GNNs), has captured the interest of scholars owing to their effectiveness and versatility in various research fields, notably in predicting vehicle trajectories in complex settings. Additionally, the literature proposes a variety of techniques to model the inter-agent interactions for vehicle trajectory prediction. One such approach involves explicitly incorporating the trajectory history of the Target Agent (TA) and its Surrounding Agents (SAs) into the model [115,116,117,118,119,120] in order to consider the impact of SAs. For instance, Dai et al. [115] proposed a two-group LSTM-based RNN approach to model the interactions between the TA and each of its neighbors and subsequently predict the future trajectory of the TA based on its trajectory history. Another approach, TrafficPredict, was introduced by Ma et al. [116], where a system architecture with two layers of LSTM recurrent units was designed to obtain the motion patterns of traffic participants and identify similar behavior among the same group of traffic participants, such as vehicles or bicycles. These methods have limitations, as they fail to account for the effect of the environment and traffic regulations on the TA’s behavior.

A potential alternative strategy for modeling social interactions among a large number of traffic participants in a given scenario involves the implementation of a social pooling mechanism [50,52,121]. This mechanism permits neighboring agents’ LSTM units to share knowledge with one another. Alahi et al. [50] proposed the S-LSTM method, which enables the recurrent units associated with SAs to connect with one another via the design of a pooling layer between each existing LSTM cell. In this technique, the hidden states are streamlined across all agents within an occupancy map. To effectively represent the interactions between all Scene Agents (SAs) in a specific setting, Gupta and colleagues [52] introduced a novel pooling approach known as S-GAN, which relies on a multi-layer perceptron (MLP) coupled with max pooling. The presented approach calculates a comprehensive pooling vector for each Temporal Attribute (TA). This vector is derived from the relative coordinates between the TA, its Spatial Attributes (SAs), and their respective hidden states. In a related work by Deo et al. [121], the authors introduced CS-LSTM, an encoder framework designed for vehicle trajectory prediction. In this approach, convolution and max pooling procedures are utilized across a spatial grid, which accurately captures the TA’s surroundings. Nevertheless, the representations obtained for the vehicles still lack integration with their individual states, leading to inefficiencies in localized computations. Messaoud et al. introduced a novel approach to tackle this problem by employing the Multi-Head Attention (MHA) pooling technique [122,123]. This technique utilizes an encoder based on LSTM to generate a vector representation for each vehicle. Then, an MHA framework is utilized to assess the interconnections among vehicles, specifically focusing on the target vehicle and its Surrounding Agents (SAs) within a defined spatial map. It has been experimentally validated that the implementation of an MHA effectively minimizes the workload of localized computations. Nevertheless, these methods’ lack of efficiency in addressing complex spatio-temporal correlations among traffic participants is a significant drawback. Additionally, the performance of these methods can be affected by the distance used to generate the occupancy grid or the number of SAs considered.

#### 2.3.2. Graph-Based Interaction Reasoning

Recently, the research area of trajectory prediction has seen a growing interest in graph-based interaction reasoning as an alternative approach to address the limitations of interaction-aware path prediction methods, as discussed in the previous section. Graph-based approaches have focused on modeling interactions between various agents within a driving scene as graphs, where nodes represent agents and edges represent inter-agent interactions. This allows for the simultaneous consideration of spatial and temporal inter-agent correlations. In a particular study, Diehl and colleagues employed a directed graph to model a highway-driving scenario. They proceeded to assess and compare the effectiveness of GAT and GCN in traffic prediction, taking into account a predetermined number of nearby vehicles [124]. In contrast, the authors’ approach to generating a homogeneous graph overlooks crucial factors such as vehicle dynamics and types. To address this, Li et al. proposed a method using a homogeneous undirected graph to capture inter-vehicle interactions and employing graph convolutions to uncover essential features within the dataset [125]. A decoder based on LSTM is utilized to predict the future trajectory of the vehicles. However, the technique still exhibits the previously mentioned constraint. Azadani et al. utilized undirected spatio-temporal graphs to model inter-vehicle interactions and analyzed the trajectory history of target vehicles and their surrounding vehicles using graph and temporal gated convolutions [126]. The future trajectory of the vehicle agents is then predicted using temporal convolutions applied to the extracted latent representations. In recent research, Wu et al. [127] proposed an encoder–decoder architecture that takes into account temporal interdependencies using Multi-Head Attention (MHA) and spatial interactions with graph attention network (GAT) modules. The resulting outputs from these separate modules are then aggregated and fed into a Long Short-Term Memory (LSTM)-based decoder. Similarly, Li et al. [90] introduced the STG-DAT system, which comprises three key modules, namely feature extraction using a multi-layer perceptron (MLP), representation extraction using a GAT as an encoder, and path generation employing Gated Recurrent Units (GRU) while considering the kinematic constraints.

Moreover, a recent study by Mo et al. introduced a directed graph model to analyze different groups of agents in a driving scenario [95]. The researchers used distinct encoders to account for the various agent types present in the scene, as each type’s specific behavior significantly influences their future trajectory patterns. Similarly, following a comparable approach, Sheng et al. developed a distance-dependent weighted graph to represent the Target Agent (TA) and the neighboring vehicles [128]. They analyzed this spatial graph using graph convolutional networks (GCN) and employed GRU units to predict the vehicles’ future trajectory. Furthermore, an alternative approach by Gao et al. involves constructing diverse sub-graphs for individual agents and a high-order graph to capture inter-agent interactions [129]. However, this method’s dense graph generation fails to account for crucial spatial and edge features among all agents. These recent advancements in modeling temporal and spatial interactions among agents have shown promising results in predicting future trajectories in complex environments.

Prior research on trajectory prediction has yielded interaction-aware approaches that are customized for particular contexts and representations. These methods often overlook certain spatial and temporal considerations or rely on dense undirected graphs to depict the inter-agent interactions. Such graphs assume that every vehicle interacts with all other surrounding agents with equal impact. In contrast, our research introduces the HSTGA model, which adopts an asymmetric social interaction reasoning approach that utilizes sparse directed graphs for both vehicles and pedestrians. This innovative model aims to address the aforementioned challenges and enhance the accuracy of trajectory prediction. Our work builds upon our previous publications [130,131,132,133,134,135,136].

## 3. Problem Definition

Assume that prior image processing has already been applied to a raw video feed to extract the position and pose of individual pedestrians and vehicles in each video frame. We assume that there are *N* pedestrians and *M* vehicles present in a video frame, represented by $p_{1},p_{2},\ldots ,p_{N}$ for pedestrians and $v_{1},v_{2},\ldots ,v_{M}$ for vehicles. The state of pedestrians $p_{i}\left(i\in \left[1,N\right]\right)$ and vehicles $v_{j}\left(j\in \left[1,M\right]\right)$ at time step *t* is denoted as follows:

(1) $$A_{obs}^{t}=\left[P_{1}^{t},P_{2}^{t},\ldots ,P_{N}^{t}\right]$$

(2) $$B_{obs}^{t}=\left[V_{1}^{t},V_{2}^{t},\ldots ,V_{M}^{t}\right]$$

where $P_{i}^{t}$ and $V_{j}^{t}$ are the lateral and longitudinal positions with the heading angles of pedestrian *i* and vehicle *j*, respectively, at time step *t*. The number of pedestrians *i* and vehicles *j* are variables in Equations (1) and (2) because different datasets/scenarios are used to evaluate this study. Equations (1) and (2) are the observed trajectories that are used as inputs to our deep learning model. $P_{i}^{t}$ and $V_{j}^{t}$ are expressed as follows:

(3) $$P_{i}^{t}=\left(x_{i}^{t},y_{i}^{t},\theta_{i}^{t}\right)$$

(4) $$V_{j}^{t}=\left(x_{j}^{t},y_{j}^{t},\theta_{j}^{t}\right)$$

In Equations (3) and (4), $x_{i}^{t}$, $x_{j}^{t}$, $y_{i}^{t}$, $y_{j}^{t}$, $\theta_{i}^{t}$, and $\theta_{j}^{t}$ are the position coordinates and the heading angles of pedestrians and vehicles at each time step *t*. The positions of the vehicles and pedestrians are relative to the world space. Using the observed trajectories $A_{obs}^{t}$ and $B_{obs}^{t}$ in the past *m* frames at time steps $t=1,\ldots ,T_{obs}$, our goal is to predict the future trajectories $A_{f}^{t}$ and $B_{f}^{t}$ several seconds into the future *h* frames at time steps $t=T_{obs}+1,\ldots ,T_{f}$ as follows:

(5) $$A_{f}^{t}=\left[P_{1}^{t+h},P_{2}^{t+h},\ldots ,P_{N}^{t+h}\right]$$

(6) $$B_{f}^{t}=\left[V_{1}^{t+h},V_{2}^{t+h},\ldots ,V_{M}^{t+h}\right]$$

## 4. Methodology

This section provides a general overview of the key components and architectural design of our multi-trajectory prediction model (HSTGA). We also delve into the specifics of each module within the framework.

### 4.1. HSTGA Overview

**Figure 3:**
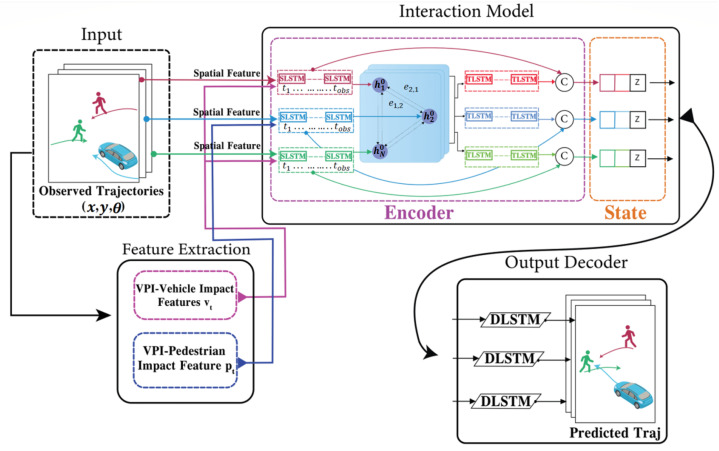
Illustration of the vehicle–pedestrian interaction model.

In order to predict the trajectories and interactions of pedestrians and vehicles within a given scene, a vehicle–pedestrian feature extraction model and a graph attention network (GAT) are employed in conjunction with two separate Long Short-Term Memory (LSTM) models, as shown in Figure 3. The first LSTM is referred to as SLSTM, where the ’S’ designates the spatial stage. The proposed SLSTM model is detailed in Section 4.3.1. It is important to note that the SLSTM discussed here is distinct from the S-LSTM introduced in [50]. This LSTM handles the individual trajectories of both vehicles and pedestrians. The GAT, situated between the SLSTM and the second model known as TLSTM, is responsible for capturing interactions between the two objects within the scene. Conversely, Temporal Long Short-Term Memory (TLSTM), where the ’T’ represents the temporal stage, is specifically designed to capture temporal interactions between vehicles and pedestrians. Both models, SLSTM and TLSTM, share the same architecture, as detailed in Section 4.3.1.

### 4.2. Vehicle–Pedestrian Interaction (VPI) Feature Extraction

**Figure 4:**
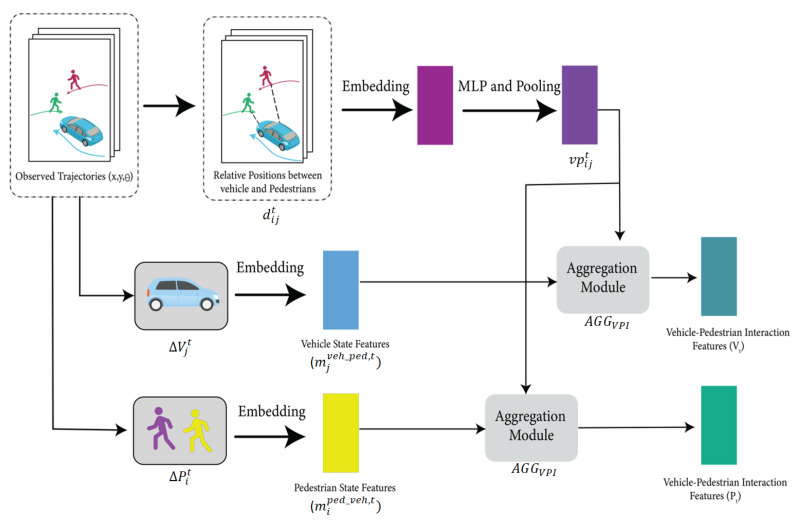
Vehicle–pedestrian interaction feature extraction model.

The interaction between vehicles and pedestrians is a significant factor in predicting their future trajectories. We build upon the work of [59,60,137,138] and implement a VPI cell into the LSTM to improve trajectory prediction by encoding vehicle–pedestrian interaction features into the individual agent LSTM. The process of extracting features related to vehicle–pedestrian interactions involves two steps, and each step has two stages, as depicted in Figure 4.

The first step extracts the vehicle–pedestrian interaction feature when considering the vehicle’s spatial influence on pedestrians. This step’s feature is then used with the pedestrian’s motion state feature (spatial feature) and is fed to the SLSTM for each pedestrian. In the first stage of this step, the interaction weights between the vehicle and pedestrian are learned using their calculated relative positions. Next, a separate embedding module is used to extract the movement state of the vehicle. Finally, the two stages are combined to obtain the features related to vehicle–pedestrian interaction, which are then fed to the SLSTM for trajectory prediction. On the other hand, the second step extracts the vehicle–pedestrian interaction feature when considering the pedestrian’s spatial influence on vehicles. The resulting feature from this step is then fed with the vehicle’s motion state (spatial feature) to the SLSTM. Stages one and two of both steps are discussed below. In stage one, the vehicle–pedestrian interaction attention weights $vp_{ij}^{t}$ between the *i*th pedestrian and the *j*th vehicle are calculated using max pooling, as shown in Equation (7).

(7) $$vp_{ij}^{t}=Pooling\left\{MLP\left(\varphi \left(d_{ij}^{t};W_{d}\right);W_{a}\right)\right\},i\in \left\{1,\ldots ,N\right\},j\in \left\{1,\ldots ,M\right\}$$

Here, Pooling (·) is the pooling layer, and MLP(·) is the multi-layer perceptron sub-network with weight $W_{a}$. Moreover, ϕ(·) is the embedding layer with weights $W_{d}$. Finally, the relative position $\left(d_{ij}^{t}\right)$ between the pedestrian and the vehicle is then calculated. Equations (3) and (4) are used to calculate the relative position using the *x* and *y* coordinates and the heading angle θ, as shown in Equation (8).

(8) $$d_{ij}^{t}=\left(x_{j}^{veh,t}-x_{i}^{ped,t},y_{j}^{veh,t}-y_{i}^{ped,t},\theta_{j}^{veh,t}-\theta_{i}^{ped,t}\right),i\in \left\{1,\ldots ,N\right\},j\in \left\{1,\ldots ,M\right\}$$

To accurately predict pedestrian trajectories, we must consider the motion state of the *j*th vehicle and then aggregate the vehicle–pedestrian interaction weights $vp_{ij}^{t}$ and the vehicle motion states $m_{j}^{veh,t}$ of the vehicle to obtain the vehicle–pedestrian interaction features or vehicle impact. We calculate the vehicle’s motion state using the equation below:

(9) $$m_{j}^{veh\_ped,t}=\phi \left(\Delta V_{j}^{t};W_{m^{veh\_ped}}\right),j\in \left\{1,\ldots ,M\right\}$$

In Equation (9), ϕ(·) represents the embedding with weights $W_{m^{veh\_ped}}$, and $\Delta V_{j}^{t}$ is the relative position of the *j*th vehicle between the current and last time steps. The final step is aggregating the vehicle–pedestrian interaction weights $vp_{ij}^{t}$ and the vehicle motion states $m_{j}^{veh\_ped,t}$ as follows:

(10) $$v_{i}^{t}=AGG_{VPI}\left(m_{j}^{veh\_ped,t},vp_{ij}^{t}\right),i\in \left\{1,\ldots ,N\right\},j\in \left\{1,\ldots ,M\right\}$$

Equation (10) is the vehicle–pedestrian interaction feature when considering the vehicle’s influence. This feature is then aggregated with the motion state of the individual pedestrian and fed to the SLSTM. For the vehicle–pedestrian interaction feature, when considering the pedestrian’s influence, the motion state of the pedestrian $m_{i}^{ped\_veh,t}$ should be calculated and then aggregated with the vehicle–pedestrian interaction weights $vp_{ij}^{t}$ to obtain the following equation:

(11) $$p_{j}^{t}=AGG_{VPI}\left(m_{i}^{ped\_veh,t},vp_{ij}^{t}\right),i\in \left\{1,\ldots ,N\right\},j\in \left\{1,\ldots ,M\right\}$$

$p_{j}^{t}$ is then aggregated with the motion state of the individual vehicle and fed to the SLSTM network. In Equations (10) and (11), $AGG_{VPI}$ represents the aggregation module stage, as shown in Figure 4.

### 4.3. Trajectory Encoding

LSTMs have been widely used to capture the motion state of pedestrians [50,56,57,60,139]. We build upon this prior work. The way an intelligent vehicle navigates through a crowded pedestrian area is, in general, similar to how human drivers do. The vehicle must consider the movements of all surrounding pedestrians and their distances from the vehicle’s trajectory. The inherent relationship between the vehicle’s movement and its proximity to the target pedestrian is a crucial factor. Moreover, the pedestrian’s motion contributes to changing the gap between them and the vehicle. This significant observation indirectly suggests that both the vehicle’s trajectory and the gap between the vehicle and the pedestrian have a significant impact on predicting the pedestrian’s trajectory. Furthermore, the pedestrian’s trajectory and their distance from the vehicle intricately affect the vehicle’s future maneuvers. Moreover, the precise prediction of forthcoming trajectories based solely on past trajectories poses a formidable challenge, primarily due to the inherent uncertainty that accompanies future trajectories, even when past trajectories are indistinguishable. To overcome this challenge, supplementary information cues, such as pedestrian intention, vehicle speed, and global scene dynamics, play a critical role in advancing the accuracy of future trajectory prediction, as these cues exhibit strong correlations with predicting pedestrian trajectories.

**Figure 5:**
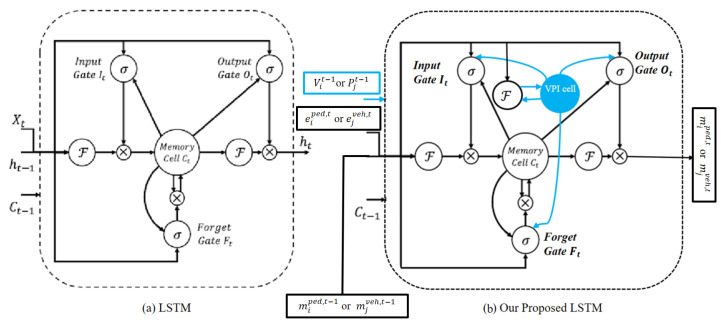
(**a**) The structure of a standard LSTM neuron. (**b**) The structure of our proposed LSTM.

Expanding on this insightful understanding and drawing inspiration from comprehensive studies [59,60,137,138], we propose the integration of an additional memory cell and dynamic rescaling of the output gate in response to changes in vehicle–pedestrian spatial interaction. We have developed a concept termed the “vehicle–pedestrian interaction (VPI) cell” to further augment the intrinsic interactions among these cues. This thoughtfully designed component aims to unravel the complex interplay between the spatial characteristics of the vehicle, the resulting changes in the pedestrian’s trajectory, and the interaction between the pedestrian’s spatial attributes and subsequent adjustments in the vehicle’s course. In our work, we propose utilizing an individual LSTM for every pedestrian and each vehicle. The architectures of the proposed Long Short-Term Memory (LSTM) and a conventional LSTM are compared in Figure 5. The initial input to the VPI cell varies based on whether the LSTM is focused on encoding the pedestrian’s or the vehicle’s trajectory. In the case of the LSTM designed for the pedestrian’s trajectory, the VPI cell’s initial input comprises a concatenation that involves gathering all the relative positions of the *j*th vehicle between the current and preceding time steps, in addition to the relative distance between the pedestrian and the vehicle as observed across frames. For a more comprehensive understanding, refer to Section 4.2 and Figure 4. With each successive time step, a new vehicle state and vehicle–pedestrian spatial features (including relative distance) are computed. Subsequently, the VPI component seamlessly integrates into the LSTM’s output gate. This strategic fusion facilitates the dynamic adjustment of the output responses, adeptly capturing alterations in the encoding of the pedestrian’s trajectory. Ultimately, the refined LSTM output ($h_{t}$) collaborates with the VPI state ($v_{i}^{t}$ or $p_{j}^{t}$), as elaborated upon in Section 4.2. These merged states then proceed to the neuron of the subsequent step, ensuring the seamless continuity of information flow.

#### 4.3.1. Pedestrian Trajectory Encoding

The implementation comprises two steps, as follows:

1. We first calculate each pedestrian’s relative position and pose to the previous time step.
  (12) $$\Delta x_{i}^{t}=x_{i}^{t}-x_{i}^{t-1}$$
  (13) $$\Delta y_{i}^{t}=y_{i}^{t}-y_{i}^{t-1}$$
  For the relative pose:
  (14) $$\Delta \theta_{i}^{t}=\theta_{i}^{t}-\theta_{i}^{t-1}$$
2. The calculated relative positions and pose are then embedded into a fixed-length vector $e_{i}^{t}$ for every time step, which is called the spatial feature of the pedestrian.
  (15) $$e_{i}^{ped,t}=\phi \left(\Delta x_{i}^{t},\Delta y_{i}^{t},\Delta \theta_{i}^{t};W_{e^{ped}}\right)$$
  where ϕ(·) is an embedding function, and $W_{e}$ is the embedding weight. This vector $e_{i}^{ped,t}$ is the input to the SLSTM cell. Then, this vector is aggregated with the vehicle–pedestrian interaction feature $v_{i}^{t}$ from Equation (10) and then fed to the SLSTM hidden state.
  (16) $$m_{i}^{ped,t}=SLSTM\left(m_{i}^{t-1},e_{i}^{t},v_{i}^{t-1};W_{m^{ped}}\right)$$
  where $m_{i}^{ped,t}$ is the hidden state of the SLSTM at time step *t*, and $W_{m^{ped}}$ is the weight of the SLSTM cell.

#### 4.3.2. Vehicle Trajectory Encoding

The methodology for encoding vehicle trajectories is identical to that of pedestrian trajectories. The following two steps are followed:

1. We first calculate each vehicle’s relative position and pose to the previous time step.
  (17) $$\Delta x_{j}^{t}=x_{j}^{t}-x_{j}^{t-1}$$
  (18) $$\Delta y_{j}^{t}=y_{j}^{t}-y_{j}^{t-1}$$
  For the relative pose:
  (19) $$\Delta \theta_{j}^{t}=\theta_{j}^{t}-\theta_{j}^{t-1}$$
2. The calculated relative positions and pose are then embedded into a fixed-length vector $e_{j}^{veh,t}$ for every time step, which is called the spatial feature of the vehicle.
  (20) $$e_{j}^{veh,t}=\phi \left(\Delta x_{j}^{t},\Delta y_{j}^{t},\Delta \theta_{j}^{t};W_{e^{veh}}\right)$$
  where ϕ(·) is an embedding function, and $W_{e}$ is the embedding weight. This vector $e_{j}^{veh,t}$ is the input to the SLSTM cell. Then, this vector is aggregated with the vehicle–pedestrian interaction feature $p_{j}^{t}$ from Equation (11) and then fed to the SLSTM hidden state.
  (21) $$m_{j}^{veh,t}=SLSTM\left(m_{j}^{t-1},e_{j}^{t},p_{j}^{t-1};W_{m^{veh}}\right)$$
  where $m_{j}^{veh,t}$ is the hidden state of the SLSTM at time step *t*, and $W_{m^{veh}}$ is the weight of the SLSTM cell.

### 4.4. Interaction Modeling and Prediction

**Figure 6:**
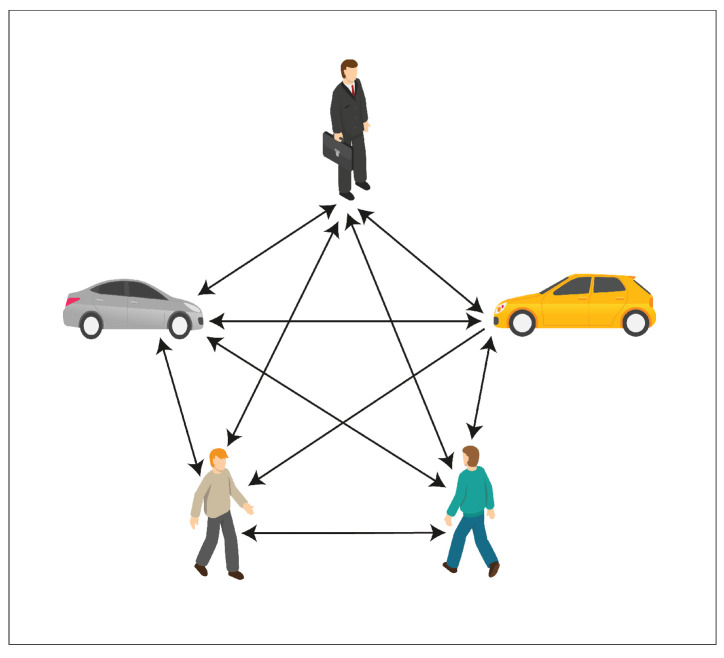
Interaction as a directed graph. Pedestrians and vehicles are nodes. The edges are the interactions between these objects.

Employing one LSTM with the VPI feature extraction model for each pedestrian and vehicle trajectory fails to capture the intricate and temporal interactions between humans and vehicles. To address this shortcoming and enable more information sharing across different pedestrians and vehicles in crowded environments, we propose treating pedestrians and vehicles as nodes on a directed graph and utilizing the recent advances in graph neural networks (GNNs). By assigning varying levels of importance to different nodes, graph attention network (GAT) models enable us to aggregate information from neighbors. Thus, we adopt a GAT as the sharing mechanism in our approach. As demonstrated in Figure 6, pedestrians and vehicles are represented as nodes in the graph, and the GAT serves as the sharing mechanism. Moreover, Figure 5 presents an illustration of the expected ways pedestrians and vehicles interact when sharing road spaces. In situations where a pedestrian or vehicle is trying to move through an environment with other moving pedestrians and vehicles, it becomes crucial for the pedestrian or vehicle to take into account all the other surrounding objects. This consideration is necessary to ensure safe movement and make correct decisions about how to effectively navigate within that specific situation.

**Figure 7:**
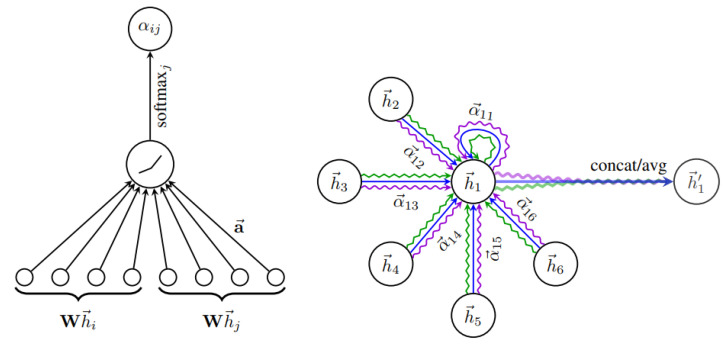
Graph attention network [140].

A graph attention network (GAT) is designed to process graph-structured data and compute node features by attending to the features of their neighboring nodes based on a self-attention mechanism [140]. Multiple graph attention layers can be stacked to form a complete GAT model [140]. A single graph attention layer is illustrated in Figure 7.

The input of the graph attention layer is $h=\vec{h_{1}},\vec{h_{2}},\ldots ,\vec{h_{NO}}$, where $\vec{h_{i}}\in R^{F},NO$ is the number of nodes, and *F* is the feature dimension of each node.

The output is $\vec{h^{{}^{\prime }}}=\vec{h_{1}^{{}^{\prime }}},\vec{h_{2}^{{}^{\prime }}},\ldots ,\vec{h_{NO}^{{}^{\prime }}}$, where $\vec{h_{i}^{{}^{\prime }}}\in R^{F^{{}^{\prime }}}$. $F^{{}^{\prime }}$ and *F* can be unequal.

In the observation period of $m_{i}^{ped,t}$ where $t=1,\ldots ,T_{obs}$ is fed to the graph attention layer. The coefficients in the attention mechanism of the node pair (i,j) can be computed by:

(22) $$\alpha_{ij}^{t}=\frac{\left(exp\left(LeakyReLU\left(a^{T}\left[W\;m_{i}^{ped,t}\|W\;m_{j}^{veh,t}\right]\right)\right)\right)}{\sum_{k\in \mathrm{NO}}exp\left(LeakyReLU\left(a^{T}\left[W\;m_{i}^{ped,t}\|W\;m_{j}^{veh,t}\right]\right)\right)}$$

where ‖ is the concatenation operation, ${\left\{\cdot \right\}}^{T}$ represents transposition, $\alpha_{ij}^{t}$ is the attention coefficient of node *j* to *i* at time step *t*, and NO represents the neighbors of node *i* on the graph. The weight matrix $W\in R^{F^{{}^{\prime }}\times F}$ is an important element in Equation (22). It represents the applied shared linear transformation of every node. The dimension of the weight matrix W is based on the dimension of the input and output of the graph attention network. *F* is the dimension of $m_{i}^{ped,t}$, and $F^{{}^{\prime }}$ is the dimension of the output. The vector $a\in R^{2F^{{}^{\prime }}}$ in Equation (22) is defined as the weight vector of a single-layer feedforward neural network. The softmax with LeakyReLU is utilized to normalize the weight vector *a*. Equation (23) defines the output of one graph attention layer for node *i* at time step *t* after normalizing the attention coefficients.

(23) $${\hat{m}}_{i}^{ped,t}=\sigma \left(\sum_{j\in \mathrm{NO}}\;\alpha_{ij}^{t}\;W\;m_{j}^{veh,t}\right)$$

In Equation (23), σ is the nonlinear function. Moreover, W is the weight matrix of a shared linear transformation from Equation (22). ${\hat{m}}_{i}^{ped,t}$, obtained following the application of two graph attention layers, incorporates the collective internal state of pedestrian *i* at time step *t*.

Moreover, the output of one graph attention layer for node *j* at *t* is given by:

(24) $${\hat{m}}_{j}^{veh,t}=\sigma \left(\sum_{j\in \mathrm{NO}}\;\alpha_{ij}^{t}\;W\;m_{j}^{veh,t}\right)$$

To capture the temporal correlations between interactions, another LSTM, called TLSTM, is used, as shown below:

(25) $$g_{i}^{ped,t}=TLSTM\left(g_{i}^{ped,t-1},{\hat{m}}_{i}^{ped,t},W_{g^{ped}}\right))$$

(26) $$g_{j}^{veh,t}=TLSTM\left(g_{j}^{veh,t-1},{\hat{m}}_{i}^{veh,t},W_{g^{veh}}\right))$$

where ${\hat{m}}_{i}^{ped,t}$ and ${\hat{m}}_{j}^{veh,t}$ are from Equations (23) and (24). $W_{g^{ped}}$ and $W_{g^{veh}}$ are the TLSTM weights for the pedestrian and vehicle, respectively, and are shared among all the sequences. In our proposed method, SLSTM is used to model the motion pattern of each pedestrian and vehicle in the scene. Moreover, another LSTM, called TLSTM, is used to model the temporal correlations of the interactions. These two LSTMs are part of the encoder structure. Then, these two LSTMs are utilized to fuse the spatial and temporal data.

At time step $T_{obs}$, there are two hidden variables $\left(m_{i}^{ped,T_{obs}},g_{i}^{ped,T_{obs}}\right)$ from two LSTMs of each pedestrian. In our implementation, these two variables are fed to two different multi-layer perceptrons, $(\delta_{1}\left(\cdot \right)$ and $\delta_{2}\left(\cdot \right))$, before being concatenated:

(27) $${\bar{m}}_{i}^{ped}=\delta_{1}\left(m_{i}^{T_{obs}}\right)$$

(28) $${\bar{g}}_{i}^{ped}=\delta_{2}\left(g_{i}^{T_{obs}}\right)$$

(29) $$h^{ped}={\bar{m}}_{i}^{ped}\|{\bar{g}}_{i}^{ped}$$

Furthermore, at each time step $T_{obs}$, there are also two hidden variables $(m_{j}^{veh,T_{obs}}$, $g_{j}^{veh,T_{obs}})$ for each vehicle. Then, these two variables are fed to two different perceptrons, $(\delta_{1}\left(\cdot \right)$ and $\delta_{2}\left(\cdot \right))$, before being concatenated:

(30) $${\bar{m}}_{j}^{veh}=\delta_{1}\left(m_{j}^{T_{obs}}\right)$$

(31) $${\bar{g}}_{j}^{veh}=\delta_{2}\left(g_{j}^{T_{obs}}\right)$$

(32) $$h^{veh}={\bar{m}}_{j}^{veh}\|{\bar{g}}_{j}^{veh}$$

Using real-world data, our goal is to simulate pedestrians’ and vehicles’ motions and the interaction between them. Three components represent the intermediate state vector of our model, namely the hidden variables of SLSTM, the hidden variables of TLSTM, and the added noise (as shown in Figure 3). The intermediate state vector is calculated as:

(33) $$d_{i}^{ped,T_{obs}}=h_{i}^{ped}\|z$$

(34) $$d_{j}^{veh,T_{obs}}=h_{j}^{veh}\|z$$

where *z* represents noise, and $h_{i}^{ped}$ and $h_{j}^{veh}$ are from Equations (29) and (32). The intermediate state vectors, $d_{i}^{ped,T_{obs}}$ and $d_{j}^{veh,T_{obs}}$, then act as the initial hidden state of the decoder LSTM (termed DLSTM). The pedestrian’s and vehicle’s predicted relative positions are shown below:

(35) $$d_{i}^{ped,T_{obs+1}}=DLSTM\left(d_{i}^{ped,T_{obs}},e_{i}^{ped,T_{obs}};W_{d^{ped}}\right)$$

(36) $$d_{j}^{veh,T_{obs+1}}=DLSTM\left(d_{j}^{veh,T_{obs}},e_{j}^{veh,T_{obs}};W_{d^{veh}}\right)$$

(37) $$\left(\Delta x_{i}^{ped,T_{obs+1}},\Delta y_{i}^{ped,T_{obs+1}},\Delta \theta_{i}^{ped,T_{obs}}\right)=\delta_{3}\left(d_{i}^{ped,T_{obs}}\right)$$

(38) $$\left(\Delta x_{j}^{veh,T_{obs+1}},\Delta y_{j}^{ped,T_{obs+1}},\Delta \theta_{j}^{veh,T_{obs}}\right)=\delta_{3}\left(d_{j}^{veh,T_{obs}}\right)$$

In Equations (35) and (36), $W_{d}$ is the weight of the Decoder Long Short-Term Memory (DLSTM). This weight plays a pivotal role in the optimization process. $e_{i}^{ped,T_{obs}}$ and $e_{j}^{veh,T_{obs}}$ are the spatial features of the pedestrian and vehicle, respectively, and are from Equations (15) and (20). In Equations (37) and (38), $\delta_{3}\left(\cdot \right)$ is a linear layer. Once the anticipated relative position at time step $T_{obs+1}$ is acquired, the DLSTM proceeds to compute the subsequent inputs. These inputs are determined by considering the most recent projected relative position, as outlined in Equation (15). Moreover, the process of translating relative positions into absolute positions, a crucial step in loss computation, can be accomplished with great simplicity. For the loss computation, we used the variety loss, as presented in reference [52]. The calculation of the variety loss is determined by following these steps. For every vehicle and pedestrian, the deep learning model generates many predicted trajectories by randomly sampling *z* from a standard normal distribution with a mean of 0 and a standard deviation of 1. Subsequently, it opts for the trajectory that exhibits the least deviation from the ground truth, using this trajectory as the model’s output for loss computation:

(39) $$L_{variety}^{ped}=\min_{k^{ped}}{∥Y_{i}-{\hat{Y}}_{i}^{k^{ped}}∥}^{2}$$

(40) $$L_{variety}^{veh}=\min_{k^{veh}}{∥Y_{j}-{\hat{Y}}_{j}^{k^{veh}}∥}^{2}$$

In Equations (39) and (40), the variables $Y_{i}$, ${\hat{Y}}_{i}^{k^{ped}}$, and $k^{ped}$ correspond to the ground-truth trajectory, the predicted trajectory, and a hyperparameter, respectively. By focusing solely on the most optimal trajectory, this particular loss function motivates the neural network to explore and encompass the range of potential outcomes aligned with the trajectory history.

## 5. Implementation Details

In our approach, training the weights of the Holistic Spatio-Temporal Graph Attention (HSTGA) trajectory prediction model involves several key components and hyperparameters to ensure effective learning. The training process aims to minimize the difference between the model’s predicted trajectories and the ground-truth trajectories from the dataset. The following steps are followed to make sure our model is performing well:

1. The variety loss is selected, as shown in Equations (39) and (40), to quantify the difference between the predicted and actual trajectories. Moreover, we used two evaluation metrics, namely the Average Displacement Error (ADE) and Final Displacement Error (FDE), to report the prediction errors.
2. The Adam optimizer is used with a good learning rate to balance fast convergence and avoid overshooting.
3. Batch-size, backpropagation, weight-update, and regularization techniques are included in our model implementation.
4. Proper datasets for training and validation are an essential part of our model implementation.
5. We monitor the performance of our model and tune the hyperparameters if needed.

The training process of our model includes fine-tuning the weights of the LSTM layers and the graph attention networks (GATs) to effectively capture vehicle–pedestrian interactions and spatio-temporal dynamics. This process progressively enhances the model’s parameters to accurately predict trajectories in complex scenarios.

In our implementation, each LSTM consists of only one layer. In Equations (15) and (20), the dimensions of $e_{i}^{ped,t}$ and $e_{j}^{veh,t}$ are set to 256, and in Equations (16) and (21), the dimensions of $m_{i}^{ped,t}$ and $m_{j}^{veh,t}$ are set to 64. The weight matrix *W* (Equation (22)) for the first graph attention layer has a dimension of 32 × 32, whereas for the second layer, it has a dimension of 32 × 64. The dimension of the attention coefficient matrix a in Equation (22) is set to 32 for the first graph attention layer and 64 for the second layer. Batch normalization is applied to the input of the graph attention layer. In Equations (25) and (26), the dimensions of $g_{i}^{ped,t}$ and $g_{j}^{veh,t}$ are set to 32. The activation function $\delta_{1}\left(\cdot \right)$ (Equations (27) and (30)) contains three layers with ReLU activation functions. The number of hidden nodes in these layers is 32, 64, and 24, respectively. Similarly, the activation function $\delta_{2}\left(\cdot \right)$ (Equations (28) and (31)) consists of three layers with ReLU activation functions, and the number of hidden nodes is 32, 64, and 16, respectively. The dimension of *z* in Equations (33) and (34) is set to 16. We trained the network using the Adam optimizer with a learning rate of 0.01 and a batch size of 64.

## 6. Experiments

### 6.1. Dataset

Datasets play a crucial role in developing and assessing deep learning models. For example, researchers frequently employ the widely used ETH [31] and UCY [32] datasets to evaluate the efficacy of pedestrian trajectory prediction models. However, these datasets are not specifically designed for urban traffic scenarios. We employed the VCI-DUT [33] and inD datasets [141] to overcome this limitation to train and evaluate our proposed HSTGA model. These datasets contain large numbers of real-world vehicle–pedestrian trajectories, encompassing various human–human, human–vehicle, and vehicle–vehicle interactions. Additionally, we compared our model against state-of-the-art pedestrian trajectory prediction models on several pedestrian datasets, including ETH, UCY, and the Stanford Drone Dataset (SDD) [142].

**Figure 8:**
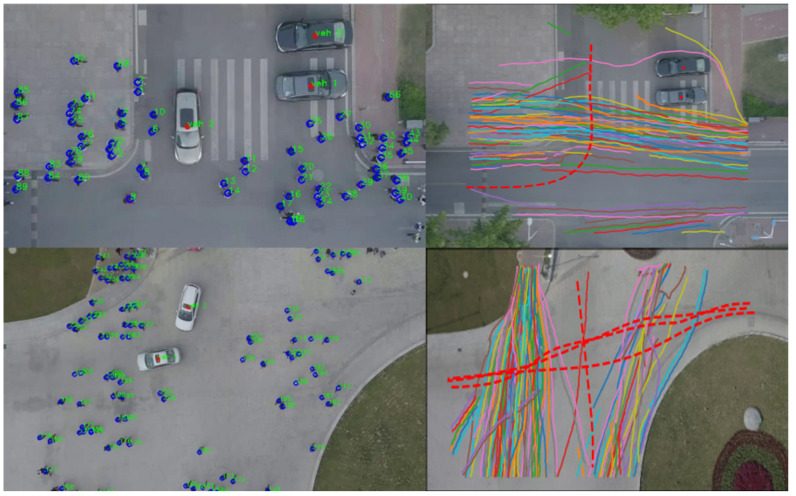
VCI-DUT Dataset with trajectories of vehicles (red dashed lines) and pedestrians (colorful solid lines). **Upper**: Intersection. **Lower**: Roundabout [33].

The VCI-DUT dataset comprises real-world pedestrian and vehicle trajectories collected from two locations on China’s Dalian University of Technology (DUT) campus, as depicted in Figure 8. The first location consists of a pedestrian crosswalk at an intersection without traffic signals, where the right of way is not prioritized for either pedestrians or vehicles. The second location is a relatively large shared space near a roundabout, where pedestrians and vehicles have free movement. Similar to the CITR dataset, the recordings were captured using a DJI Mavic Pro Drone equipped with a downward-facing camera, which was positioned high enough to go unnoticed by pedestrians and vehicles. The footage has a resolution of 1920 × 1080 with a frame rate of 23.98 fps. The dataset primarily comprises trajectories of college students leaving their classrooms and regular cars passing through the campus. The dataset comprises 17 clips of crosswalk scenarios and 11 clips of shared-space scenarios, including 1793 trajectories. Some of the clips involve multiple VCIs, i.e., more than two vehicles simultaneously interacting with pedestrians, as illustrated in Figure 8.

**Figure 9:**
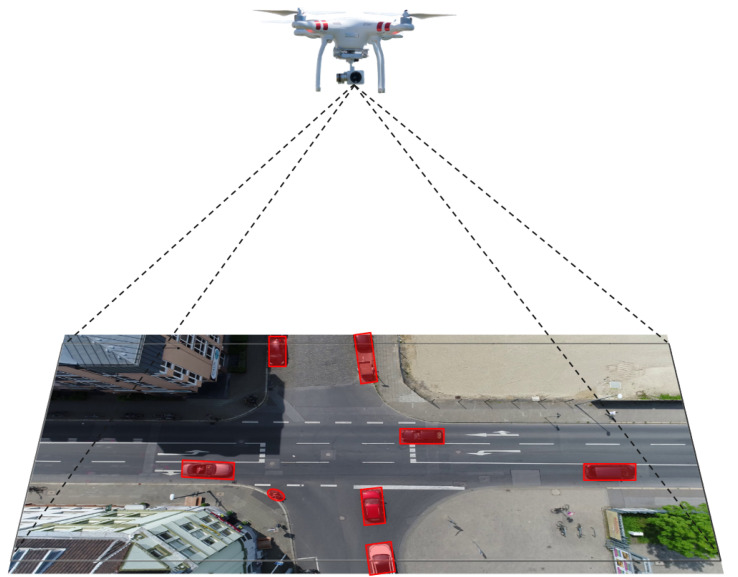
inD dataset [141].

The second dataset utilized in this study is the inD dataset, as depicted in Figure 9. This new dataset contains naturalistic vehicle trajectories captured at intersections in Germany. Traditional data collection methods are prone to limitations such as occlusions; however, by using a drone, these obstacles are overcome. Traffic at four distinct locations was recorded, and the trajectory for each road user was extracted, along with their corresponding type. State-of-the-art computer vision algorithms were used to obtain positional errors, typically less than 10 cm. The inD dataset is applicable to numerous tasks, including road-user prediction, driver modeling, scenario-based safety validation of automated driving systems, and data-driven development of highly automated driving (HAD) system components.

### 6.2. Evaluation Metrics

Following prior works [50,56,57,60,139], we used two error metrics to report prediction errors:

- Average Displacement Error (ADE): The mean distance between the actual and predicted trajectories over all predicted time steps, as specified in Equation (40).
- Final Displacement Error (FDE): The mean distance between the actual and predicted trajectories at the last predicted time step, which is expressed in Equation (41).

(41) $$ADE_{ped}=\frac{\sum_{i\in N}\sum_{t=T_{obs}+1}^{T_{f}}{∥Y_{i}^{ped,t}-{\hat{Y}}_{i}^{ped,t}∥}^{2}}{N\times (T_{f}-T_{obs})}$$

(42) $$FDE_{ped}=\frac{\sum_{i\in N}{∥Y_{i}^{ped,t}-{\hat{Y}}_{i}^{ped,t}∥}^{2}}{N},t=T_{f}$$

In Equations (41) and (42), *N* is the number of pedestrians. To find the ADE and FDE for vehicles, *N* is replaced with *M*, which is the number of vehicles.

## 7. Results and Analysis

### 7.1. Quantitative Results

Our model has been extensively trained and evaluated using two datasets: the VCI-DUT dataset and the inD dataset. The VCI-DUT dataset consists of 17 video clips that effectively portray crosswalk scenarios and an additional 11 video clips that depict shared-space scenarios. To ensure optimal model performance, a training subset of 10% from the VCI-DUT dataset was utilized, whereas the remaining portion was exclusively employed for rigorous model evaluation. It is noteworthy to mention that the training subset predominantly encompasses intersection scenarios, focusing on the intricate dynamics between pedestrians and vehicles in such settings.

However, it is important to highlight that our model was intentionally not trained on roundabout scenarios. This decision was based on the recognition of the heightened complexity and increased interaction complexity between pedestrians and vehicles observed in roundabouts. By excluding roundabout scenarios from the training process, we aimed to evaluate the generalization capability of our model, specifically in the context of previously unseen and intricate scenarios, such as roundabouts. By conducting an in-depth evaluation of the proposed Holistic Spatio-Temporal Graph Attention (HSTGA) model in roundabout settings, we aim to provide valuable insights into its generalization capabilities and further contribute to the advancement of pedestrian–vehicle interaction research.

Moreover, we trained our model on additional datasets, including ETH, UCY, HOTEL, ZARA1, and ZARA2. We also used 40% of the dataset for training and the remainder for evaluation. We started the investigation by evaluating our model on the pedestrian-only dataset. The ADE and FDE results (in meters) for 12 time-step predictions are shown in Table 1; lower results are better. The bold font represents the best results. The proposed model outperformed the previous approaches, such as Social-LSTM [50], Social Attention [143], Social-GAN [136], CIDNN [57], STGAT [56], and Step Attention [37], in both the ADE and FDE. The results demonstrate that the use of human–human, human–vehicle, and vehicle–vehicle information improves the accuracy of pedestrian trajectory predictions.

Table 2 presents a comparative analysis of the factors that influence pedestrian trajectory in LSTM-based models and our proposed method. We investigated the influence of the social interaction (SI), the pedestrian–vehicle interaction (VPI), and different inputs, including the relative position (RP), the relative velocity (RV), and learning the vehicle–pedestrian interaction adaptively (LIA).

In Table 3, we demonstrate the evaluation outcomes of our method on the VCI-DUT and inD datasets and compare them with baseline techniques, including state-of-the-art DNN-based pedestrian prediction methods.

- Constant Velocity (CV) [79]: The pedestrian is assumed to travel at a constant velocity.
- Social GAN (SGAN) [52]: A GAN architecture that uses a permutation-invariant pooling module to capture pedestrian interactions at different scales.
- Multi-Agent Tensor Fusion (MATF) [54]: A GAN architecture that uses a global pooling layer to combine trajectory and semantic information.
- Off-the-Sidewalk Predictions (OSP) [79]: The probabilistic interaction model introduced in [79].

As shown in Table 3, the proposed HSTGA method outperformed previous works in both the shared spaces of the DUT dataset and the unsignalized intersections of the inD dataset.

### 7.2. Qualitative Results

**Figure 10:**
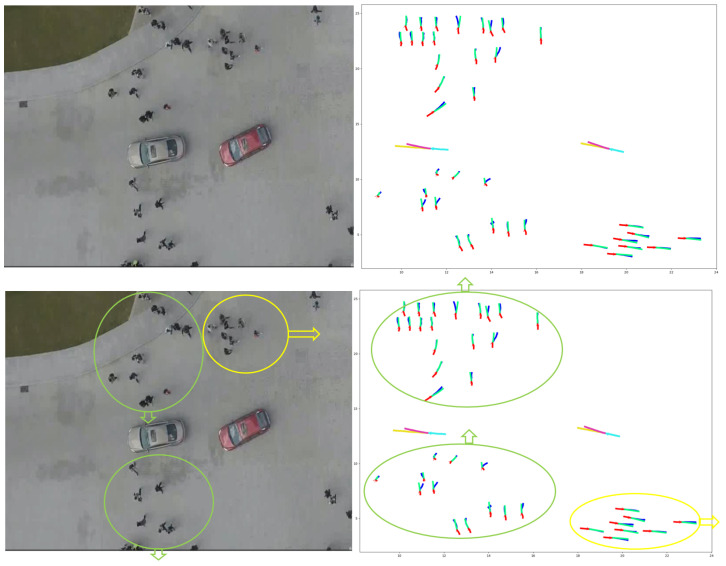
The output trajectories of the model on the 1st scenario of the DUT dataset. **Left**: Visual of the scene. **Right**: Trajectory model and prediction. Pedestrians: red (observed trajectory), blue (ground truth), and green (predicted trajectory). Vehicles: turquoise (observed trajectory), yellow (ground truth), and pink (predicted trajectory). In the lower image, objects distinguished by a specific color are enclosed within a drawn outline and an arrow that indicates the direction of movement.

**Figure 11:**
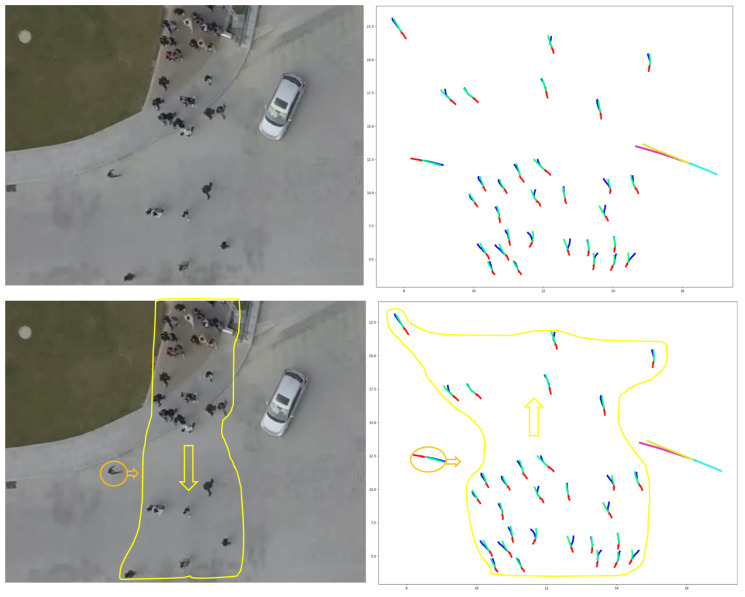
The output trajectories of the model on the 2nd scenario of the DUT dataset. **Left**: Visual of the scene. **Right**: Trajectory model and prediction. Pedestrians: red (observed trajectory), blue (ground truth), and green (predicted trajectory). Vehicles: turquoise (observed trajectory), yellow (ground truth), and pink (predicted trajectory). In the lower image, objects distinguished by a specific color are enclosed within a drawn outline and an arrow that indicates the direction of movement.

**Figure 12:**
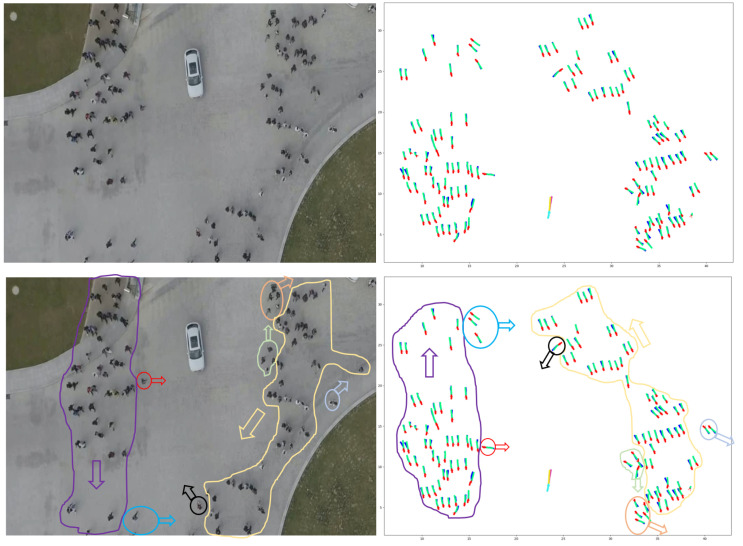
The output trajectories of the model on the 3rd scenario of the DUT dataset. **Left**: Visual of the scene. **Right**: Trajectory model and prediction. Pedestrians: red (observed trajectory), blue (ground truth), and green (predicted trajectory). Vehicles: turquoise (observed trajectory), yellow (ground truth), and pink (predicted trajectory). In the lower image, objects distinguished by a specific color are enclosed within a drawn outline and an arrow that indicates the direction of movement.

**Figure 13:**
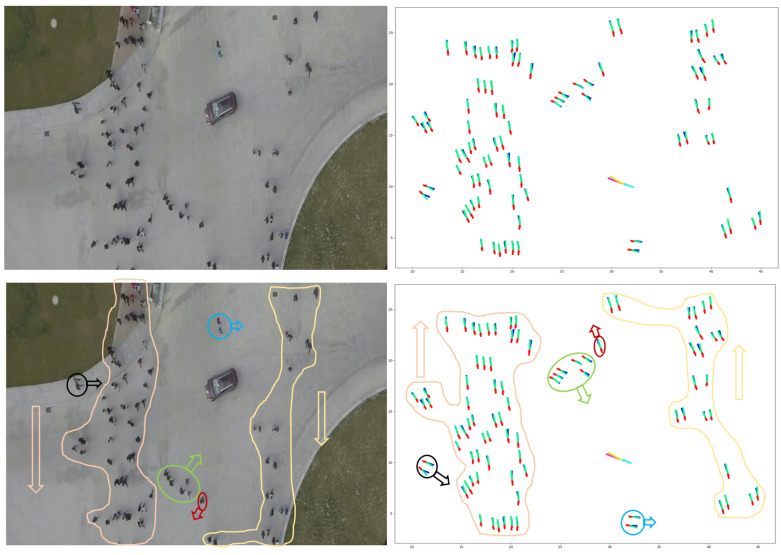
The output trajectories of the model on the 4th scenario of the DUT dataset. **Left**: Visual of the scene. **Right**: Trajectory model and prediction. Pedestrians: red (observed trajectory), blue (ground truth), and green (predicted trajectory). Vehicles: turquoise (observed trajectory), yellow (ground truth), and pink (predicted trajectory). In the lower image, objects distinguished by a specific color are enclosed within a drawn outline and an arrow that indicates the direction of movement.

**Figure 14:**
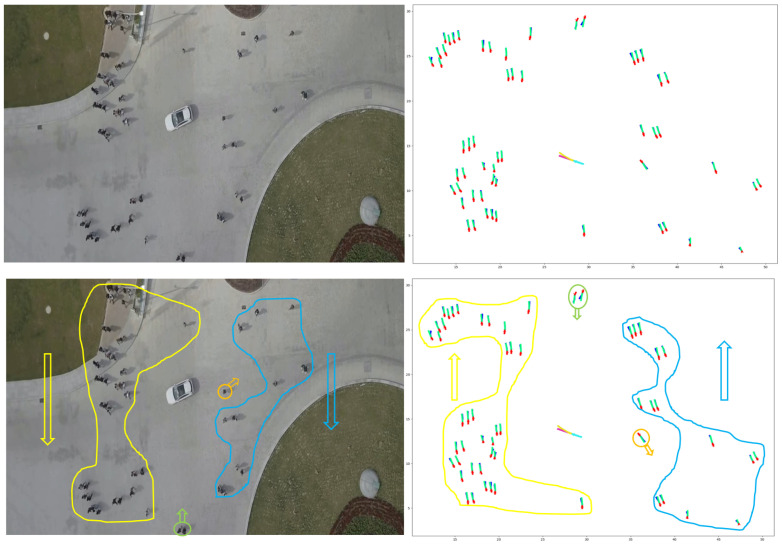
The output trajectories of the model on the 5th scenario of the DUT dataset. **Left**: Visual of the scene. **Right**: Trajectory model and prediction. Pedestrians: red (observed trajectory), blue (ground truth), and green (predicted trajectory). Vehicles: turquoise (observed trajectory), yellow (ground truth), and pink (predicted trajectory). In the lower image, objects distinguished by a specific color are enclosed within a drawn outline and an arrow that indicates the direction of movement.

To qualitatively analyze the performance of the HSTGA model, we plotted the predicted trajectories against the ground truth. The following scenarios (Figure 10, Figure 11, Figure 12, Figure 13 and Figure 14) show the qualitative results of our model, where pedestrians interact with vehicles in a very challenging environment. The background images presented in Figure 10, Figure 11, Figure 12, Figure 13 and Figure 14 are screenshots extracted from the raw video. The video itself has a resolution of 1920 × 1080 pixels and operates at a frame rate of 23.98 frames per second (fps). It is important to note that the coordinates of the image extracted from the video (left image) are in terms of image pixels, whereas the predicted trajectories of the image (right image) are on a scale of meters.

**Figure 15:**
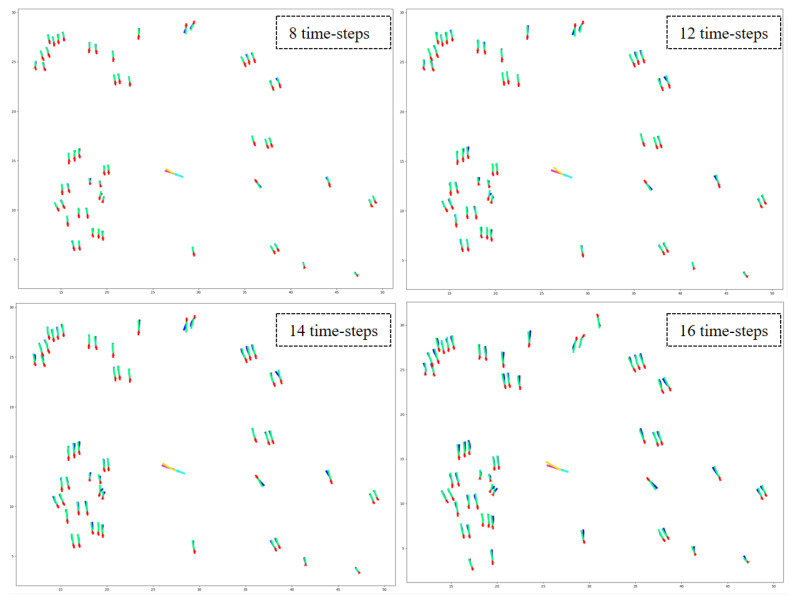
Predicted trajectories at 8, 12, 14, and 16 time steps. Pedestrians: red (observed trajectory), blue (ground truth), and green (predicted trajectory). Vehicles: turquoise (observed trajectory), yellow (ground truth), and pink (predicted trajectory).

**Figure 16:**
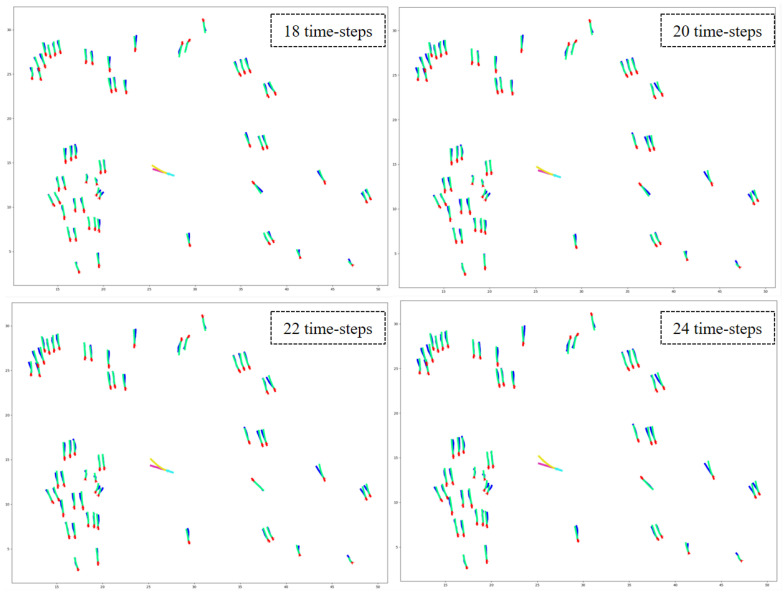
Predicted trajectories at 18, 20, 22, and 24 time steps. Pedestrians: red (observed trajectory), blue (ground truth), and green (predicted trajectory). Vehicles: turquoise (observed trajectory), yellow (ground truth), and pink (predicted trajectory).

Our investigation involved a rigorous evaluation of the predictive capacities of our model, which entailed the prediction of future outcomes across a range of distinct time steps. Specifically, we examined the predictive accuracy at 8, 12, 14, 16, 18, 20, 22, and 24 time steps ahead. These chosen time steps were critical in assessing the model’s efficacy in forecasting future events. To illustrate our findings, we present the following figures (Figure 15 and Figure 16) that offer a comprehensive depiction of the obtained results for each designated time step. Importantly, the data presented in these figures pertain specifically to the 5th scenario, ensuring a focused and contextually relevant analysis.

The experimental results in Figure 15 and Figure 16 demonstrate the capability of our model in long-term trajectory prediction. These figures serve as empirical evidence, substantiating the claim that our model exhibits remarkable efficacy in predicting trajectories over extended time periods. Notably, our findings reveal that the accuracy of long-term predictions, spanning 16, 18, 20, 22, and 24 time steps, is on par with that of short-term predictions covering 8 time steps. This signifies the robustness and reliability of our model’s predictive capabilities across varying temporal horizons.

## 8. Conclusions

In this study, we propose a novel encoder–decoder interaction model named Holistic Spatio-Temporal Graph Attention (HSTGA) for trajectory prediction in vehicle–pedestrian interaction. HSTGA aims to predict long-horizon pedestrian and vehicle trajectories by modeling pedestrian–vehicle interactions in non-signalized and non-crosswalk scenarios. The proposed model uses a trajectory-based approach to capture the complex interactions between pedestrians and vehicles. HSTGA integrates a holistic spatio-temporal graph attention mechanism that learns the attention weights of the spatial and temporal features of pedestrians and vehicles. The proposed method outperforms state-of-the-art pedestrian trajectory prediction models on various benchmark datasets, highlighting the effectiveness of the HSTGA model. In order to effectively capture the interaction features between pedestrians and vehicles, a vehicle–pedestrian interaction feature extraction model that utilizes a multi-layer perceptron (MLP) sub-network and max pooling has been proposed. The MLP sub-network is responsible for extracting the features of both pedestrians and vehicles, whereas the max pooling operation aggregates these features into a single vector. The extracted features are then input into an LSTM network to predict the trajectories of both pedestrians and vehicles. This feature extraction model enhances the model’s ability to capture the intricate interactions between pedestrians and vehicles, resulting in heightened prediction accuracy. Compared to other methods, the proposed approach reduces both computational and data requirements, rendering it suitable for real-time applications. The MLP sub-network extracts features in parallel, reducing the overall time complexity of the model. The max pooling operation combines the features of pedestrians and vehicles into a single vector, thereby decreasing the number of input parameters required for the LSTM network. Furthermore, the proposed approach solely utilizes the historical trajectories of pedestrians and vehicles, thus eliminating the need for external data sources. Extensive evaluations conducted on diverse datasets containing numerous challenging scenarios involving the interactions between vehicles and pedestrians demonstrate the effectiveness and efficiency of the proposed approach.

## Figures and Tables

**Table 1:** Quantitative results of all the baseline models and our model (in bold). Two evaluation metrics, namely the ADE and FDE, are presented (lower results are better).

Metric	Dataset	LSTM	S-LSTM [50]	Social Attention [143]	CIDNN [57]	SGAN [52]	STGAT [56]	HSTGA (Ours)
ADE	ETH	0.70/1.09	0.73/1.09	1.04/1.39	0.89/1.25	0.60/0.87	0.56/0.65	**0.42/0.53**
ADE	HOTEL	0.55/0.86	0.49/0.79	1.95/2.51	1.25/1.31	0.48/0.72	0.27/0.35	**0.22/0.31**
ADE	UNIV	0.36/0.61	0.41/0.67	0.78/1.25	0.59/0.90	0.36/0.60	0.31/0.51	**0.27/0.44**
ADE	ZARA1	0.25/0.41	0.27/0.47	0.59/1.01	0.29/0.50	0.21/0.34	0.21/0.34	**0.19/0.31**
ADE	ZARA2	0.31/0.52	0.33/0.56	0.55/0.88	0.28/0.51	0.27/0.42	0.20/0.29	**0.20/0.27**
FDE	ETH	1.45/2.41	1.48/2.35	1.83/2.39	1.89/2.32	1.19/1.62	1.10/1.12	**0.96/1.03**
FDE	HOTEL	1.17/1.91	1.01/1.76	2.97/2.91	2.20/2.36	0.95/1.61	0.50/0.66	**0.44/0.52**
FDE	UNIV	0.77/1.31	0.84/1.40	1.56/2.54	1.13/1.86	0.75/1.26	0.66/1.10	**0.55/0.98**
FDE	ZARA1	0.53/0.88	0.56/1.00	1.24/2.17	0.59/1.04	0.42/0.69	0.42/0.69	**0.41/0.62**
FDE	ZARA2	0.65/1.11	0.70/1.17	1.09/1.75	0.60/1.07	0.54/0.84	0.40/0.60	**0.38/0.61**

**Table 2:** Interaction and influencing factors of LSTM-based models and our model (in bold).

Model Name	Dataset	ADE	FDE	Influencing Factors						
LSTM	ETH	0.70/1.09	1.45/2.41	-	-	-	-	-	-	-
S-LSTM [50]	ETH	0.73/1.09	1.48/2.35	SI	-	RP	-	-	-	-
SocialAttention [143]	ETH	1.04/1.39	1.83/2.39	SI	-	RP	-	-	-	-
CIDNN [78]	ETH	0.89/1.25	1.89/2.32	SI	-	RP	-	-	-	-
SGAN [52]	ETH	0.60/0.87	1.19/1.62	SI	-	RP	RV	-	-	-
STGAT [56]	ETH	0.56/0.65	1.10/1.12	SI	TI	RP	RV	-	-	-
**HSTGA (Ours)**	ETH	**0.42/0.53**	**0.96/1.03**	**SI**	**TI**	**RP**	**RV**	**LIA**	-	**HA**

SI: spatial interaction; TI: temporal interaction; RP: relative position; RV: relative velocity; VPI: vehicle–pedestrian interaction; VVI: vehicle–vehicle interaction; HA: heading angle; LIA: learning vehicle–pedestrian interaction adaptively using LSTM.

**Table 3:** Quantitative results on DUT and inD datasets.

Metric	Dataset	CV [79]	SGAN [52]	MATF-S [54]	OSP [79]	HSTGA (Our)
ADE	DUT	0.39	0.62	1.65	0.22	**0.11**
FDE	DUT	0.38	0.66	1.87	0.30	**0.16**
ADE	inD	0.50	0.98	1.01	0.42	**0.23**
FDE	inD	0.50	1.09	1.12	0.50	**0.29**

## Data Availability

Two datasets are used namely VCI-DUT and inD datasets. The VCI-DUT data is available in a publicly accessible repository at [https://github.com/dongfang-steven-yang/vci-dataset-dut, accessed on 19 June 2023]. The inD dataset is available upon request at [https://www.ind-dataset.com/, accessed on 19 June 2023].
